# Supplementary figures and images for: Automated Western immunoblotting detection of anti-SARS-CoV-2 serum antibodies
Source: Eur J Clin Microbiol Infect Dis. 2021 Mar 3;40(6):1309–17. doi: 10.1007/s10096-021-04203-8 (PMC7928199; doi:10.1007/s10096-021-04203-8)

a-

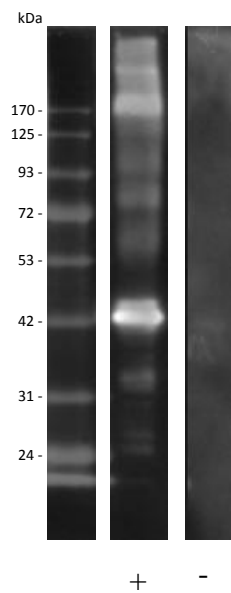

b-

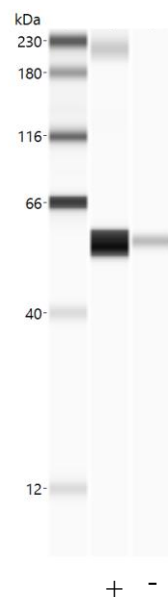

c-

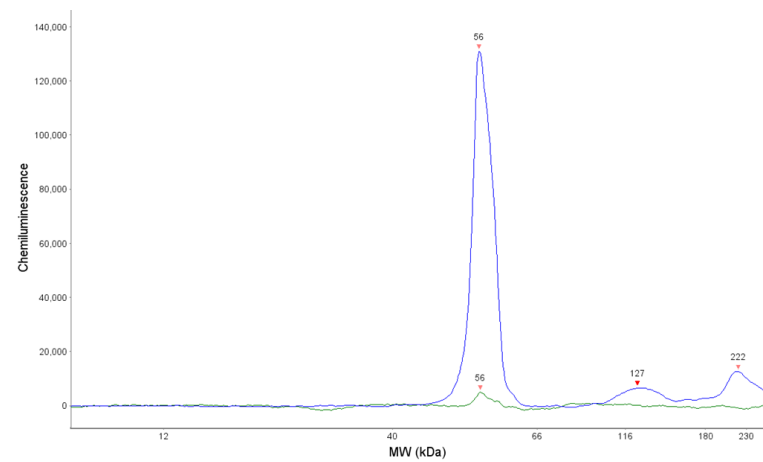

Supplement: Supplementary file 1 — Strips of conventional western immunoblotting (a) and lane view of automated Western immunoblotting (b) incubated with serum collected from one COVID-19 positive patient quoted “+” and serum collected from one non COVID-19 patient quoted “-“. The first lane represents the molecular mass marker in kDa. (c) chromatogram of chemiluminescence intensity detected by JessTM Simple Western in the capillaries on positive (blue) and negative (green) sera. (PDF 132 kb) [file 10096_2021_4203_MOESM1_ESM.pdf]
